# Compassion-Focused Group Therapy for Treatment-Resistant OCD: Initial Evaluation Using a Multiple Baseline Design

**DOI:** 10.3389/fpsyg.2020.594277

**Published:** 2021-01-12

**Authors:** Nicola Petrocchi, Teresa Cosentino, Valerio Pellegrini, Giuseppe Femia, Antonella D’Innocenzo, Francesco Mancini

**Affiliations:** ^1^Department of Economics and Social Sciences, John Cabot University, Rome, Italy; ^2^Compassionate Mind Italia, Rome, Italy; ^3^Scuola di Psicoterapia Cognitiva S.r.l., Rome, Italy; ^4^Department of Social and Developmental Psychology, Faculty of Medicine and Psychology, Sapienza University of Rome, Rome, Italy; ^5^Department of Psychological Sciences, Guglielmo Marconi University, Rome, Italy

**Keywords:** compassion-focused therapy, obsessive–compulsive disorder, fear of guilt, self-reassuring, self-criticism, multiple baseline design, self-compassion, compassionate mind training

## Abstract

Obsessive–compulsive disorder (OCD) is a debilitating mental health disorder that can easily become a treatment-resistant condition. Although effective therapies exist, only about half of the patients seem to benefit from them when we consider treatment refusal, dropout rates, and residual symptoms. Thus, providing effective augmentation to standard therapies could improve existing treatments. Group compassion-focused interventions have shown promise for reducing depression, anxiety, and avoidance related to various clinical problems, but this approach has never been evaluated for OCD individuals. However, cultivating compassion for self and others seems crucial for OCD patients, given the accumulating research suggesting that fear of guilt, along with isolation and self-criticism, can strongly contribute to the development and maintenance of OCD. The primary aim of this pilot study was to evaluate the acceptability, tolerability, and effectiveness of an 8-week group compassion-focused intervention for reducing OCD symptoms, depression, fear of guilt and self-criticism, and increasing common humanity and compassionate self-reassuring skills in treatment-resistant OCD patients. Using a multiple baseline experimental design, the intervention was evaluated in a sample of OCD patients (*N* = 8) who had completed at least 6 months of CBT treatment for OCD, but who continued to suffer from significant symptoms. Participants were randomized to different baseline assessment lengths; they then received 8 weekly, 120-min group sessions of compassion-focused therapy for OCD (CFT-OCD), and then were tested again at post-treatment and at 1 month follow up. Despite the adverse external circumstances (post-treatment and follow-up data collection were carried out, respectively, at the beginning and in the middle of the Italian lockdown due to the COVID-19 pandemic), by the end of treatment, all participants demonstrated reliable decreases in OCD symptoms, and these improvements were maintained at 4-week follow-up for seven of eight participants. The intervention was also associated with improvements in fear of guilt, self-criticism, and self-reassurance, but less consistent improvements in depression and common humanity. Participants reported high levels of acceptability of and satisfaction with the intervention. Results suggest that the intervention may be beneficial as either a stand-alone treatment or as an augmentation to other treatments.

## Introduction

Obsessive–compulsive disorder (OCD) is a debilitating mental health condition characterized by obsessions (persistent and distressing thoughts, images, doubts, or urges) and compulsions (interfering and ritualistic mental or physical behaviors the individual feels compelled to perform in order to alleviate distress and/or prevent negative outcomes; American Psychiatric Association APA, 2013), which affects 2–3% of the population (Brakoulias et al., 2017). OCD has a high comorbidity with depression and anxiety disorders and can easily develop into a treatment-resistant condition. Although effective treatments for OCD exist, such as cognitive–behavior therapy (CBT) that includes exposure and response prevention (ERP), only about half of patients seem to benefit from them when treatment refusal and dropout rates are taken into account (e.g., Fisher and Wells, 2005). This constitutes a limit of the actual treatments of OCD, given that patients who attain only partial recovery are less likely to maintain their treatment improvements (Simpson et al., 2004), and their quality of life is negatively impacted by residual symptoms (Fontenelle et al., 2010; Key et al., 2017).

It has been suggested that one of the reasons for this sub-optimal capacity to treat OCD is that, traditionally, CBT treatments have mainly focused on intrapersonal/cognitive determinants of the disease (i.e., the role of negative appraisal in the development and persistence of obsessions), while overlooking the importance of social/relational elements (such as the participant’s relationships with themselves and others) and self-conscious moral emotions such as guilt. Indeed, guilt has long been considered an important aspect of the phenomenology of OCD. Even if OCD individuals do not seem to differ from anxious controls in levels of trait or state guilt (Steketee et al., 1991), there is increasing empirical evidence suggesting that OCD patients are characterized by heightened *fear of guilt* and that obsessive activity is aimed at preventing, reducing, or neutralizing the possibility of being guilty (see Shapiro and Stewart’s, 2011). Parental psychological control based on guilt induction over matters one could not control, harsh criticism, and withdrawal of love, support, and validation in response to child wrongdoing seems to play a significant role on the future genesis of OCD, by rendering the child increasingly fearful of guilt and motivated to atone for it (Barcaccia et al., 2015; Mancini, 2019). Indeed, retrospective accounts of childhood experiences of guilt induction significantly predicted feelings of mental contamination and washing rituals in non-clinical individuals (Berman et al., 2012).

Moreover, in the last decade, empirical evidence supporting the distinction between two types of guilt has accumulated. One might feel the emotion of guilt based on compassionate/altruistic principles without the transgression of moral norms (i.e., altruistic guilt), or based on the transgression of moral norms without the presence of actual victims (i.e., deontological guilt; Mancini, 2019). Intriguingly, it is the deontological type of guilt, and *not* the altruistic/compassionate guilt, that is associated with the feeling of disgust, consequently with more washing behaviors (the Lady Macbeth effect), and also is the type experienced (and dreaded) more by OCD patients (D’Olimpio and Mancini, 2014; Ottaviani et al., 2019).

Only a few studies have tested the effectiveness of psychological interventions intended to directly impact fear of guilt, and most have used cognitive procedures like socratic dialogue, cognitive restructuring, and double standard, in order to detoxify maladaptive beliefs about guilt (Cosentino et al., 2012; Perdighe and Mancini, 2012). However, mounting empirical evidence suggests that therapeutic “experiential” approaches that focus on increasing compassion for oneself and others, such as compassion-focused therapy (CFT; Gilbert, 2014; Kirby et al., 2015), may be extremely effective in helping OCD patients allow and accept the possibility of experiencing guilty feelings and respond more adaptively to their fear of guilt.

Compassion-focused therapy proposes a model of affect regulation involving three cross-regulating evolved emotional systems: the threat and self-protection system, the drive and resource-seeking system, and the soothing system. Some emotional difficulties, such as high shame, self-criticism and, in the context of OCD, the fear and avoidance of some emotional experience such as guilt, can be conceptualized as stemming from a threat system that has been defensively hyperactivated by interpersonal traumas such as bullying or emotionally abusive dynamics in the family of origin. However, CFT conceptualizes another cause of emotional difficulties as an under-activated soothing system. Adults who have had experiences of parental warmth and affection are able to regulate threat-focused emotions by activating soothing memories, emotions or schemas of support, encouragement, and validation (Gilbert, 2017; Naismith et al., 2019). Indeed, Mancini et al. (2004) found that OCD patients evaluated the facial expressions of anger, contempt, and disgust they imagined were aimed at them as more severe than expressions of fear, sadness, and joy. Furthermore, they remembered being the target of these expressions during childhood more than controls.

Compassion-focused therapy, from its evolutionary perspective, sees compassion as a motivational system rooted in mammalian caring and defines it as “the sensitivity to suffering in self and others, with a commitment to try to alleviate and prevent it” (Gilbert, 2017). For individuals with OCD, particularly when treatment resistant, cultivating compassion may address pervasive feelings of isolation, “abnormality,” unworthiness and insufficiency, while increasing common humanity and countering the tendencies to withdraw and self-criticize. Rather than relying primarily on higher-level reason and logic to challenge distorted cognitions about oneself, compassion may activate the attachment and caregiving emotion-regulation system, creating a felt sense of inner safeness and caring (Gilbert and Procter, 2006). Research has shown that compassion is associated with increased heart rate variability (HRV), an index of the vagal regulation of the heart and the ability to downregulate physiological arousal when facing stress (for a recent meta-analysis see Di Bello et al., 2020). Moreover, CFT interventions, whose primary aim is to cultivate all three flows of compassion (from others, to others and to the self), have been shown to increase the ability to generate a self-validating and self-soothing response to our own suffering, while reducing self-critical self-talk (Boersma et al., 2015). Cultivating a compassionate attitude toward others, especially in a group context, might help the OCD individuals feel less “alone,” “abnormal,” and ashamed of their suffering (Cândea and Szentagotai-Tãta, 2018), an important outcome given the difficulties in mentalizing and empathizing with the negative emotional experiences the OCD patients present (Pino et al., 2016). Moreover, by cultivating the ability to soothe, validate, and compassionately comfort themselves when distress surfaces, OCD patients might experience less fear of guilt and be more willing to accept this unpleasant emotional state (“I am only human, like everyone, I can’t avoid doing mistakes, and if I make them I’m motivated to be there for myself, trying to forgive me”), and therefore more able to abstain from compensatory defensive behaviors (compulsions). A deeper and “experiential” understanding of the origin and functions of their self-criticism might reduce the shameful, punishing self-attacking that OCD patients exert on themselves as a way to keep their compulsion under control, which usually leads to the exacerbation of symptoms, adding to the morbidity of their condition (Mancini, 2019).

Preliminary findings offer some support to the usefulness of a compassion-focused intervention in the treatment of OCD patients. Two recent studies on OCD patients demonstrated that self-compassion was correlated to reduced OCD symptoms and that this correlation was partially explained by lower emotion regulation difficulties (Chase et al., 2019; Eichholz et al., 2020). Another recent cross-sectional study comparing treatment-seeking adults (*N* = 1871) and non-treatment-seeking adults (*N* = 540) found that participants with clinically significant OCD symptoms reported lower trait mindfulness and self-compassion compared to participants with clinically significant anxiety/depression and to non-clinical controls, with mindfulness and self-compassion as unique predictors of OCD symptoms, even when controlling for depression severity (Leeuwerik et al., 2020).

Moreover, mindfulness-based interventions such as mindfulness-based cognitive therapy (MBCT), which indirectly promote a compassionate and accepting attitude toward internal experience, have been found to be effective in alleviating OCD symptoms (Key et al., 2017; Didonna et al., 2019). However, to the best of our knowledge, no study so far has developed and tested a compassion-focused intervention that is entirely aimed at helping OCD patients cultivate a less self-critical and more compassionate attitude toward themselves and others. This is important, considering that a recent meta-analysis on 25 randomized trials has found significant beneficial effects when CBT was integrated with psychosocial augmentations (especially with patients with more severe OCD), while other forms of augmentations (for example, the combination of CBT with d-cycloserine or serotonin reuptake inhibitors or mindfulness-based augmentations of CBT) were not significantly more effective than CBT alone (Guzick et al., 2018).

Thus, the primary aim of this pilot study was to evaluate the acceptability, tolerability, and effectiveness of an 8-week group compassion-focused intervention for reducing OCD symptoms, depression, fear of guilt and self-criticism, and increasing common humanity and compassionate self-reassuring skills in treatment-resistant OCD patients. The intervention (CFT-OCD) was an adaptation of group CFT for OCD: it included an emphasis on developing greater understanding and acceptance of the “unchosen nature” of our evolved brain, of its “loops,” and cultivating present moment awareness and compassion to intrusive obsessive thoughts to help participants disengage from unhelpful automatic responses (compulsions).

Multiple baseline, a type of single-case experimental design that randomizes individuals to different lengths of baseline phase before starting treatment, was used as a time- and cost-effective method for evaluating effectiveness while controlling for the passage of time and repeated assessments. In particular, it helps to differentiate between a genuine treatment effect and natural recovery over time or other confounding factors. As a primary outcome, it was hypothesized that OCD symptoms would remain stable or increase during the baseline phase, be decreased at the end of the treatment, and remain low during follow-up. As secondary outcomes, decreases in depressive symptoms, fear of guilt and self-criticism, and increases in compassionate self-reassuring and common humanity were expected to occur during the treatment phase. Finally, we predicted that participants would report that they were satisfied with the CFT intervention.

## Materials and Methods

### Participants

**FIGURE 1:**
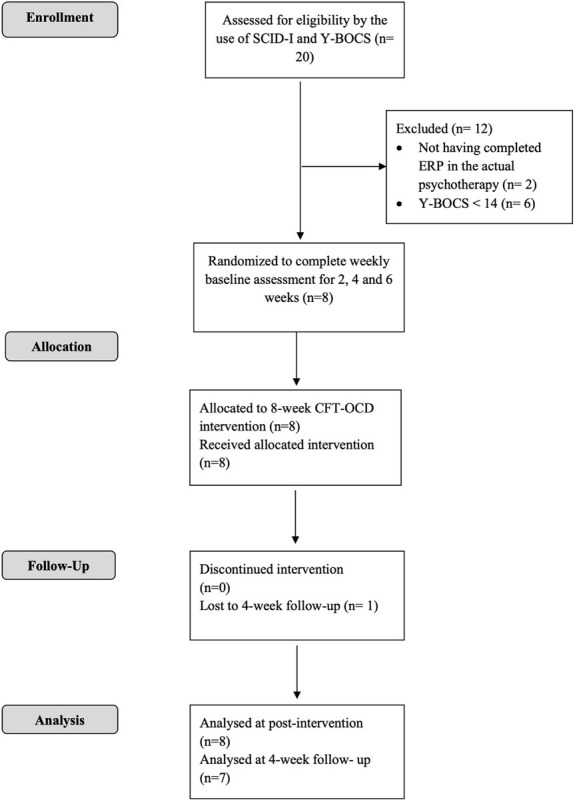
Consolidated Standards of Reporting Trials (CONSORT) diagram.

Participants were recruited among patients treated at the Unit for treatment of anxiety and mood disorders of the School of Cognitive Psychotherapy (SPC) in Rome. The procedures were approved by the ethic committee of the University Guglielmo Marconi in Rome. Participants were recruited from patients who, in the current therapy, had completed at least 6 months of CBT treatment for OCD (with ERP), referred to us by their treating clinicians. Referred patients were subsequently contacted by phone to further describe the study and to invite them to the initial assessment session. The intervention started once the group reached eight participants, which has been described as the optimal number of members for a group intervention (Yalom, 2008). Participants did not receive any monetary compensation at the end of their participation in the study. Inclusion criteria for the study were as follows: being over 18 years of age; having a principal diagnosis of OCD, as diagnosed by an expert clinician using the Structured Clinical Interview for Diagnosis (SCID-I; First et al., 1994); having completed at least 6 months CBT with ERP; reporting significant residual OCD symptoms at the end of CBT (defined as a score on the Y-BOCS greater than or equal to 14; Storch et al., 2015); not having previous experience with compassion-focused practices. Exclusion criteria included the following: significant mental or physical comorbid disorders or illnesses (lifetime or current bipolar I or II disorder, schizophrenia, delusional disorder, brain injuries, post-traumatic stress disorder or physical illnesses); alcohol or drug abuse or dependence within the last 6 months; a current marked risk to self (self-harm or suicide). Significant physical and medical comorbidities were assessed by a semi-structured face-to-face interview conducted by the same clinician who administered the SCID-I. Taking psychotropic medication was not an exclusion criterion in this study; for those taking a psychoactive medication, eligibility required no changes in dose during the 3 months prior to entering study and maintenance of a stable dose for duration of the study. In multiple baseline designs, the baseline phase can be used to exclude participants who spontaneously recover before the beginning of the treatment. Participants were required to show stable or worsening symptomatology during baseline, defined in this study as no more than a 10-point decrease on either the Y-BOCS or OCI-r between the last two baseline observations. The Consolidated Standards of Reporting Trials (CONSORT) diagram (see Figure 1) summarizes the process of recruitment and flow of participants through the study. Through screening, 20 patients were assessed for eligibility; 12 were excluded for not meeting inclusion criteria; 8 completed the intervention and the post-intervention assessment; 7 completed the 4-week follow-up assessment. All participants reported being single, except for P5 that reported being in a stable relationship. Participant characteristics are presented in Table 1.

**TABLE 1:** Participants’ characteristics.

	**Age**	**Gender**	**Education**	**Type of DOC**	**Medications**	**Age of OCD onset**	**Years of therapy since onset**	**Previous treatments**	**Number of CBT/ERP sessions before CFT treatment**
P1	36	F	Bachelor’s degree	D and C	No	Adolesc.	>1	CBTstandard	40
P2	24	M	Bachelor’s degree	U and R, C and W	Anafranil	Childhood	5	CBT standard	29
P3	38	F	Bachelor’s degree	C and W, D and C	Fluoxetine	18 years	>1	CBTstandard	43
P4	41	F	Master’s degree	C and W, S and C, U and R	Elopram	Childhood	16	CBT standard Psychoan.	42
P5	31	F	Master’s degree	U and R, D and C	No	30 years	>1	CBTstandard Schema therapy	36
P6	29	M	Bachelor’s degree	C and W	Escitalopram	21 years	>1	CBTstandard	33
P7	34	M	Bachelor’s degree	D and C	No	32 years	>1	CBTstandard	37
P8	31	M	Master’s degree	U and R, D and C	No	23 years	>1	CBTstandard	32

*D and C, doubts about accidental harm and checking; U and R, unacceptable taboo thoughts and mental rituals; C and W, contamination and washing; S and C, symmetry, arranging, and counting.*

### Procedure and Study Design

To evaluate the effectiveness of the group CFT-OCD intervention, a randomized, multiple baseline design was used (Kazdin, 2011; Smith, 2012). After the initial screening for eligibility, informed consent was obtained by all participants, and they were randomized to different baseline assessment lengths (T1 and T2—between 2 and 6 weeks). During the subsequent treatment phase, all participants received 8 weekly, 120-min group sessions of CFT-OCD. Participants completed the full battery of measures at baseline (T1), pre-treatment (T2), post-treatment (T3), and 1 month after the end of the treatment (T4). Given the pilot nature of the study, and in order to avoid the assessment fatigue that OCD patients often experience as result of their meticulousness and obsessive intrusive thoughts about exactness (Roth et al., 2004), participants were not assessed during the acute treatment phase. Randomizing participants to baseline periods of varying lengths enables assessment of whether symptom changes occur only when the intervention is applied. This design allows causal inferences to be made and controls for many threats to internal validity, including the passage of time and repeated assessments. Each participant acts as their own control; thus, fewer participants are needed to demonstrate change as a result of the intervention. The multiple assessments also provide information on whether symptom changes coincide with the introduction of the treatment.

### Measures

#### Yale–Brown Obsessive–Compulsive Scale

Yale–Brown Obsessive–Compulsive Scale (Y-BOCS; Goodman et al., 1989; Italian version by Hénin, 2012) was used to assess severity of OCD symptoms. It is a widely used clinician-administered interview for assessing the presence and the severity of OCD symptoms in adults. The Y-BOCS includes two sections: the Symptom Checklist (Y-BOCS-SC) and the Severity Scale (Y-BOCS-SS). The Severity Scale, which has been used in the present study, consists of 10 items that assess the severity of obsessions (five items) and compulsions (five items) using a five-point Likert-type scale (from 0 to 4; the total score of the scale may range between 0 and 40). In the present study, the total Y-BOCS-SS score and the obsessions and compulsions subscales are reported. The scale showed strong internal consistency for the total score and each subscales (α = 0.89–93; Melli et al., 2015).

#### Obsessive–Compulsive Inventory-Revised

Obsessive–Compulsive Inventory-Revised (OCI-R; Foa et al., 2002; Italian version by Sica et al., 2009) is a brief, 18-item, self-report questionnaire designed to measure obsessive–compulsive symptom presence and distress on a five-point Likert scale from 0 (“not at all disturbed”) to 4 (“extremely disturbed”). The OCI-R assesses symptoms on six different dimensions including washing, checking, ordering, obsessing, hoarding, and mental neutralizing (three items each). The Italian version of the OCI-R has been found to have good internal consistency (α = 0.85). In the present study, only the total score was computed.

#### Beck Depression Inventory

Beck Depression Inventory (BDI−II; Beck et al., 1996; Italian version by Sica and Ghisi, 2007) is a 21−item self−report inventory designed to measure the presence and intensity of depressive symptomatology in both adult and adolescent populations. The questionnaire is presented in multiple choice format with each item scored on a four−point scale ranging from 0 (low intensity) to 3 (high intensity) with overall total scores ranging from 0 to 63. The inventory has demonstrated excellent internal consistency reliability (e.g., α = 0.91–0.93; Beck et al., 1996).

#### Fear of Guilt Scale

Fear of Guilt Scale (FOGS; Chiang et al., 2016; Italian version by Cosentino et al., 2020) is a 17-item scale designed to determine the extent to which respondents fear feeling guilt and behave in ways to minimize, prevent, or atone for guilt on a seven-point Likert scale and consists of two factors—Punishment (drive to punish oneself or atone for guilt, inability to forgive oneself) and Harm Prevention (belief that one can and should be able to prevent guilt). The FOGS is closely linked to OCD symptoms and has demonstrated strong internal consistency and convergent, divergent, and concurrent validity. Only the total score, which has shown good internal consistency (α = 0.92; Chiang et al., 2016), has been employed in the present study.

#### The Forms of Self-Criticizing/Attacking and Self-Reassuring Scale

The Forms of Self-Criticizing/Attacking and Self-Reassuring Scale (Gilbert et al., 2004; Italian version by Petrocchi and Couyoumdjian, 2016) was used to evaluate how individuals “treat themselves” when things go wrong. This instrument is composed of 22 items with a five-point Likert scale from 0 (not at all like me) to 4 (extremely like me) and consists of three subscales: *self-criticizing*, which evaluates feelings of inadequacy and senses of irritation and frustration toward the self; *self-attacking*, which evaluates a more extreme form of self-criticism, characterized by feelings of self-repugnance and desires to hurt the self in response to failures and setbacks; *self-reassuring*, which evaluates the capacity to be self-soothing and consider the self with kindness and compassion. Adequate levels of internal consistency (α ranging from 0.76 to 0.91) and construct validity were found for all the subscales in the original and in the Italian version (Petrocchi and Couyoumdjian, 2016). In this study, *self-criticizing* and *self-attacking* subscales were combined, as recommended by recent investigations on the factor structure of the scale (Halamová et al., 2018). Higher scores at these subscales indicate higher levels of self-criticism and self-reassurance, respectively.

#### Common Humanity

Common humanity subscale of the Self-compassion Scale (Neff, 2003; Italian version by Petrocchi et al., 2014) was used to assess the participants’ ability to remind themselves that suffering is part of the human nature, and to see one’s experiences as part of the larger human experience rather than as separating and isolating. Common humanity subscale contains four items, with a five-point Likert scale ranging from 1 (*Almost Never*) to 5 (*Almost Always*), and was obtained calculating the mean of the items’ scores. The subscale has demonstrated good internal consistency (α = 0.81).

#### Acceptability

At post-intervention, participants were asked to quantitatively rate the usefulness (from 1 = not at all useful to 7 = extremely useful) of several aspects of the intervention: the CFT-DOC psychoeducation, the group climate, visualizations techniques, body techniques, meditation techniques, practices in pairs, and practices at home. They were also asked to rate (from 1 = not at all to 5 = extremely) how acceptable the intervention was, how satisfied they were with it, whether they would recommend this treatment to friends or family suffering from OCD, and how much the treatment had improved their quality of life. Participants also provided qualitative feedback on some of the changes they had noticed as a result of the intervention and suggestions on how to improve the treatment.

### Intervention—Compassion-Focused Therapy for OCD (CFT-OCD)

Treatment was delivered by two facilitators. The first facilitator (NP) is a psychotherapist with more than 8 years of direct training with Paul Gilbert and one of the co-authors of the soon-to-be published manual for group CFT. The second facilitator (AD) is a psychotherapist trained in CFT, for more than 5 years, by the first author, with extensive experience in leading mindfulness meditation interventions. Both facilitators have practiced personal meditation for over 5 years. All sessions were conducted in the group therapy room at the counseling center of the School of Cognitive Psychotherapy (SPC) in Rome. Participants attended 8 weekly sessions of 120 min of group CFT. Treatment rationale and objectives, the structure of the sessions, and the scripts of the compassion-focused visualizations and meditations were based on the soon-to-be published manual for group CFT (one the co-authors of the manual is the first author of the present paper) and the book *Mindful compassion* (Gilbert and Choden, 2014). All group sessions had the following format: (1) initial 10-min “landing” meditation, (2) review and discussion of the previous week’s home practice with a focus on exploring the barriers and difficulties that clients faced, (3) introduction of a specific CFT-related theme and practice of relevant CFT exercises within the group, (4) assignment of home practice for clients to practice over the subsequent week, and (5) final compassion-focused mediation. All meditations and visualizations were audio-recorded and uploaded on a shared online group. At the beginning, participants were asked to complete a home-practice log reporting the CFT meditations and tools they had used during the week. However, the extreme scrupulousness, exactness, and self-doubt that characterized OCD patients made this task too anxiety inducing for participants, and it was decided by the group to discontinue it, opting for a less structured review and discussion of the previous week’s home practice at the beginning of each session.

Compassion-focused therapy for OCD began with psychoeducation on the evolved nature and difficulties of the human mind such as tendencies for negativity bias, negative rumination, aversion of certain emotions and experiences, shame, and self-criticism. In particular, patients were introduced to the concept of “tricky brain” and to the nature of the evolved human mind, its emphasis on threat, and how this can create unhelpful loops between thoughts, feelings, and behaviors. They were invited to reflect on the fact that built from genes they did not choose, and that genetic background constituted one of the reasons not only for physical, but also for psychological suffering (there is prevalence of OCD diagnoses among first-degree relatives of persons affected with the same pathology; Fyer et al., 2005). Humans also grew up in environments that that they did not choose, which, in their case, have established the foundation of their vulnerability to OCD. Clients were nudged to consider and reflect in group how interpersonal context, family atmosphere, and certain styles of childrearing (e.g., rigid obsessive beliefs in parents, especially tied to inflated responsibility, overestimation of threats, perfectionism, and intolerance of uncertainty) might have played a role in the emergence of the obsessive symptomology they had developed later on. In fact, family climate is often described by OCD patients as rigid and characterized by a marked attention to morality and normative behavior. It is not rare for OCD patients to remember harsh and scornful reproaches and the interruption of affective bonds (often accompanied by a peculiar facial expression, represented by a “long face”) as punishment, without explicit forgiveness during childhood (Shapiro and Stewart, 2011). Such serious perceived threats could have been motivators for behaving impeccably, a typical goal of the obsessive mind. This psychoeducational aspect was aimed to help clients adopt a de-shaming and depersonalizing approach, which created the context for realizing that much of what goes on in their “obsessive” mind (their doubts, their fear of experiencing certain emotions, such as guilt, their compulsive behaviors) is not their fault, but that it is their responsibility to learn how to work with the mind in a way that is helpful. Patients were then offered experiential insights into the nature of three emotion regulation systems (the threat, the seeking, and the soothing systems), and the regulating effect that the soothing system, via increased activity of the vagus nerve and the prefrontal cortex, exerts on the other two systems. They were helped to focus on their OCD difficulties and their self-criticism as stemming from their threat system (i.e., in terms of safety behaviors) and to explore the usefulness of becoming understanding and compassionate to those safety behaviors (e.g., to de-shame and de-pathologize, activating the soothing system). Patients were taught to explore the fears and the functions behind their self-criticism and to identify some of the protective purposes it may be trying to serve (e.g., preventing the client from being scolded, rejected, or humiliated for their mistakes and for their OCD symptoms). In general, obsessive patients typically show a primary self-critical rumination (“how could I have been so careless, couldn’t I have thought ahead of time about avoiding such contact? I did something really stupid!”) which triggers compulsions, which are usually followed by a secondary self-critical rumination (“I’m crazy! It’s not rational. I’m strange. I’m ruining my life and my parents’ life! I have to stop!”). Patients were guided to realize that both self-critical self-talks triggered the threat system, with unintended psychophysiological consequences that maintain OCD (reduced prefrontal inhibitory regulation, increased selective attention to potential threats, increased self-evaluation moral standards, “more better than sorry” reasoning style, avoidance etc.; Gilbert, 2019). They then were invited to experience, with several body-based visualization and mediation practices, how extending warmth and compassion to others as much as to the suffering parts of themselves (including their self-criticism) had beneficial effects in terms of lowering arousal and in particular, dampening self-criticism and its “looping effect” on OCD symptomatology. It was very important that the participant understood the way external and internal soothing and reassurance can stimulate soothing systems, and viewed CMT as a kind of physiotherapy for the brain. Table 2 describes the content addressed in each CFT group session.

**TABLE 2:** Overview of group sessions.

**Session topic**	**Key elements**
1. Introducing compassion	Group exploration of compassion: definition, intro to fears of compassion; influences of evolved brain, genetics, and social context on OCD vulnerability. Introduction to the nature, functions, and unintended consequences for OCD of the self-critical voice; why and how compassion might help when we experience OCD symptoms; introductions and outlining of group objectives.
2. The three emotion regulation systems	Three emotion regulation systems and how they are unbalanced in OCD; obsessions and compulsions as stemming from the threat system. Definition of internal (fear of guilt) and external threats for an OCD patient. How to use soothing rhythm breathing, friendly facial expression, and friendly inner tone of voice to start stimulating the soothing system and heart rate variability.
3. Mindfulness and attention	How to use attention intentionally for awareness and amplification; how mindfulness can facilitate less reactivity to obsessive thoughts and feelings, especially when it is infused with compassionate intention (compassionate labeling); compassionate refocusing; compassionate body posture
4. Safeness vs. safety and the first flow of compassion	Discussion on the difference between safety and safeness and how crucial affiliation with self and others is to generate a state of safeness; explorations of the “power of visualization”; compassion from others: “safe place” imagery and “compassionate other” imagery and how they can create safeness (instead of safety) when they experience obsessive doubts.
5. Compassionate self and the second flow of compassion (for others)	“Compassionate self” imagery: focusing on oneself as a compassionate person with the three core qualities of compassion: wisdom, strength and authority, and commitment; discussion on how they can bring compassion to life difficulties that other people in their life might experience, starting with the other OCD group members; discussion of what it would be like to deliberately cultivate this self and operate from this part of self more often when obsessive thoughts emerge. Visualizations of future scenarios of their compassionate self-dealing with triggering circumstances
6. Self-criticism	Exploration of the forms and functions of self-criticism—purpose and unintended effects on our OCD symptoms; using “compassionate self” imagery to address both primary (“Am I stupid?? I should have been more careful!!”) and secondary (“I can’t go on like this, I need to stop compulsions, I’m crazy!”) self-criticism; developing a repertoire of compassionate statements and behaviors to use in response to the self-critical voice.
7. The third flow of compassion: compassion for our multiple selves	Visualization: exploring multiple emotions of the threat system (primary guilt and the avoidance of it) and their conflicts; addressing multiple emotions and their needs through our compassionate self; discussion of common traps and barriers, and practice within the group.
8. Cultivating self-compassion and wrap-up	Cultivating self-compassion; compassionate letter writing to themselves; review and relapse prevention; discussion of lessons learned, experiences people want to take away from group and plans for moving forward

### Data Analyses

In multiple baseline single case series design, visual inspection is one of the main methods employed to describe data and make inferences about the changes’ reliability due to a treatment. Such method consists of the visual examination of graphed data in order to evaluate the amount and rate of changes across phases (e.g., baseline, treatment, and follow-up). Given the reduced sample size that often characterizes single-case studies, visual inspection is considered a conservative and reliable approach with respect to other classic statistical tests. Indeed, it relies on consistent effects that are readily seen (Kazdin, 2011). In this study, Y-BOCS, OCI-r, FOGS, and BDI-II data were plotted graphically in order to examine changes in OCD-related symptoms and constructs for each participant. Likewise, common humanity subscale (CHS) and self-criticism and self-reassurance (FSCRS) data were graphically examined as compassion-related measures.

Auxiliary to the visual inspection, non-overlap methods for analyzing the difference between phases in single-case designs were implemented. These methods approach the problem of trying to quantify differences between two adjacent phases in a single-case study by descriptively summarizing the extent to which data points in the phases do not overlap. Parker et al. (2011) recommended two methods as the most robust combination: the percentage of all non-overlapping data (PAND) and non-overlap of all pairs (NAP). These indexes express the percentage of participants’ scores at post-treatment and follow up that don’t overlap (in the expected direction) with participants’ scores at baseline. Moreover, the percentage of data points exceeding the median (PEM) should also be considered, as it is least likely to be influenced by autocorrelation (Manolov et al., 2010). Therefore, these three non-overlap methods have been employed in the study. For both visual inspection graphs and overlap statistics, the R package SCAN was implemented using the RStudio graphical interface (RStudio Team, 2015).

We also computed a change score and the related 95% confidence interval for each participant on all the measures included in the study. This method was used to supplement the visual inspection in order to monitor the symptoms’ variations across particular time intervals. Specifically, change scores on each outcome measure were calculated to assess change from first baseline to pre-treatment (i.e., last baseline), change from pre- to post-treatment, and change from pre-treatment to follow-up (see Table 3 for variables and means used to calculate reliability coefficients). Then, standard error of the difference was computed (*S*_*diff*_; Jacobson and Truax, 1991), which embodied the average variation in score that would be anticipated on that measure by chance variation alone, between two detection times. *S*_*diff*_ was computed by using the SDs and reliability coefficients (i.e., Cronbach’s alpha) of the scales at the first baseline measurement time (see Supplementary Table 1). Afterward, *S*_*diff*_ for each measure was multiplied by the *Z* critical value of 1.96 to create a 95% CI around each participant’s change score. This CI provides the range of plausible values for each change score within a 95% confidence level; additionally, when the CI does not include zero, the observed change can be considered a reliable change (Jacobson and Truax, 1991).

**TABLE 3:** Descriptive statistics of variables at the first baseline measurement time.

	**Y-BOCS**	**Obses.**	**Comp.**	**OCI-r**	**FOGS**	**BDI-II**	**CHS**	**S-C**	**S-R**
*M*	2.80	2.90	2.70	1.55	5.27	0.87	2.17	2.46	1.48
*SD*	0.61	0.52	0.77	0.58	0.53	0.38	0.76	0.75	0.90
*Alpha*	0.93	0.79	0.96	0.75	0.69	0.79	0.90	0.75	0.91
*S*_*diff*_	0.23	0.34	0.22	0.41	0.42	0.24	0.34	0.34	0.38
*95% CI*	±0.45	±0.66	±0.42	±0.80	±0.82	±0.47	±0.67	±0.66	±0.75

*YBOCS, Yale–Brown Obsessive–Compulsive Scale, and the obsessive (Obses.) and compulsive (Comp.) sub-dimensions; OCI-r, Obsessive–Compulsive Inventory Revised; FOGS, Fear of Guilt Scale; BDI-II, Beck Depression Inventory II; CHS, Common Humanity Subscale; S-C, Self-Criticism; S-R, Self-Reassurance. These indices were used to compute change scores.*

Given the relatively small number of participants, we tried to provide further support and robustness to our results through another non-parametric test. Thus, we implemented a Wilcoxon Signed Ranks Test for the non-parametric comparison of the Y-BOCS, OCI-r, BDI-II, FOGS, CHS, and FSCRS scores between the baseline and post-treatment, as well as between the baseline scores and the follow-up. Moreover, all test statistics were associated with an effect size (*r*). These effect sizes were computed by dividing the *Z* test statistic by the square root of the total number of observations (Fritz et al., 2012).

Finally, an overall and standardized mean difference was calculated for each dependent variable, using a Hedges’ *g* effect size specifically developed for single-case designs (Shadish et al., 2014). The standardized differences pertained to the comparisons of the average score at the baseline phases in respect to post-treatment and follow-up. This effect size index is particularly suitable for single-case studies since it takes into account the scores’ autocorrelation across the different measurement times and corrects the estimate for small sample bias. Moreover, this effect size can be incorporated in a future meta-analysis study as it is based on classical Cohen’s *d* metric. Following the procedure outlined by Shadish et al. (2014), a test of the statistical significance of *G* against the two-tailed critical value of *Z* and the related 95% confidence interval was computed for all the interested standardized differences. Specifically, the authors suggest to compute the square root of the *G*’s variance to obtain the associated standard error and to multiply the latter by the *Z* critical value of 1.96 to obtain a 95% CI. These analyses were run using the SPSS macro developed by Shadish (2015).

## Results

### Visual Inspection, Non-overlapping Data Analyses, and Change Score

**FIGURE 2:**
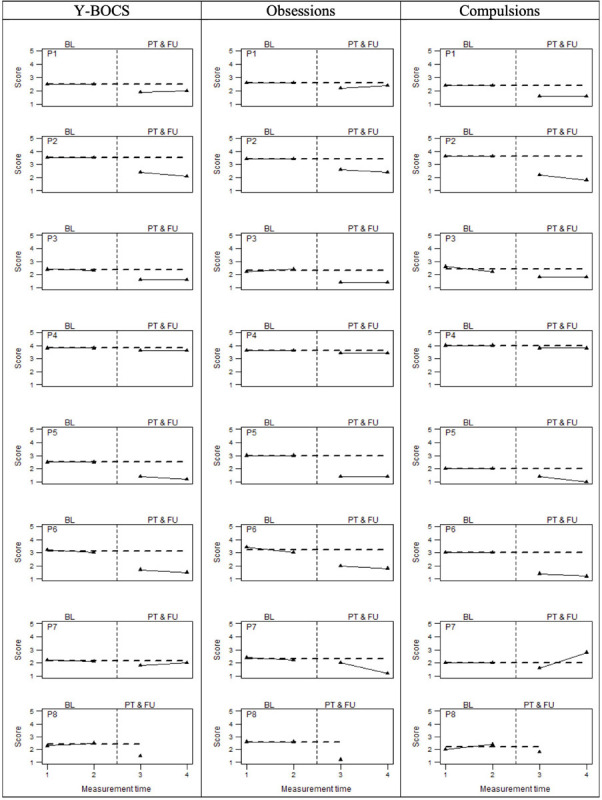
OCD symptoms severity (Y-BOCS total score and sub-dimensions) at baseline (BL), Post-treatment (PT), and follow-up (FU). The perpendicular dotted line represents the intervention.

As regards the primary outcome, Figure 2 graphically displays the OCD-related symptoms severity assessed by means of the Y-BOCS. Specifically, it shows the total score and the specific sub-dimensions (compulsions and obsessions) for all eight participants. Visual inspection suggested that the scores at the post-treatment phases were all lower than those at both the baseline phases, except for the compulsions subscale score of participant 7 at the follow-up measurement time. This visual evidence was corroborated by the non-overlapping data analyses (Table 4), which showed only a little overlap of data points at the Yale–Brown compulsion scores. Indeed, PAND, NAP, and PEM were all equal to 100% for the obsessive dimension and the overall score of Y-BOCS, whereas around 93% for the compulsions dimension. This means that for all participants, total Y-BOCS and the obsession subscale scores at post-treatment and follow-up were lower than scores at baseline. These results indicate large beneficial intervention effects. Consistently, it highlights a reliable improvement of all participants on the overall Y-BOCS, comparing the pre-treatment phase with those of post-treatment and follow-up (see Supplementary Table 1). A similar trend is found when we look at the Yale–Brown’s sub-dimensions: all participants reported a reliable improvement on the compulsions dimension of the Y-BOCS after the treatment. A unique exception was participant 7, who demonstrated a reliable worsening at the follow-up. However, the same participant showed a reliable improvement on the obsessions dimension at the same phase. Regarding the obsessions dimension, the exception was instead participant 4, who did not show any improvement across phases, but showed a reliable improvement on the compulsions dimension. Moreover, most of the participants did not report a reliable change score during the two baseline intervals, further pointing out the treatment effect. The only exception was participant P6, who showed a slight but reliable improvement during the two pre-treatment assessments (T1 and T2). However, the participant was still retained in the analysis, given that the scores on the OCI-r did not corroborate such improvement and the Y-BOCS improvement was less than a 10-point decrease.

**TABLE 4:** Non-overlapping data analyses.

**Measures**	**PAND**	**NAP**	**PEM**
Yale–Brown Total Score	100%	100%	100%
Yale–Brown Obsessions Score	100%	100%	100%
Yale–Brown Compulsions Score	93.5%	93.7%	93.7%
Obsessive–Compulsive Inventory Revised	85.5%	85.9%	87.5%
Fear of Guilt Scale	80.6%	81.2%	93.7%
Beck Depression Inventory II	66.1%	70.2%	68.7%
Common Humanity	74.2%	73.5%	75%
Self-Criticizing/Attacking	87.1%	87.5%	87.5%
Self-Reassuring	77.4%	82.9%	81.2%

**FIGURE 3:**
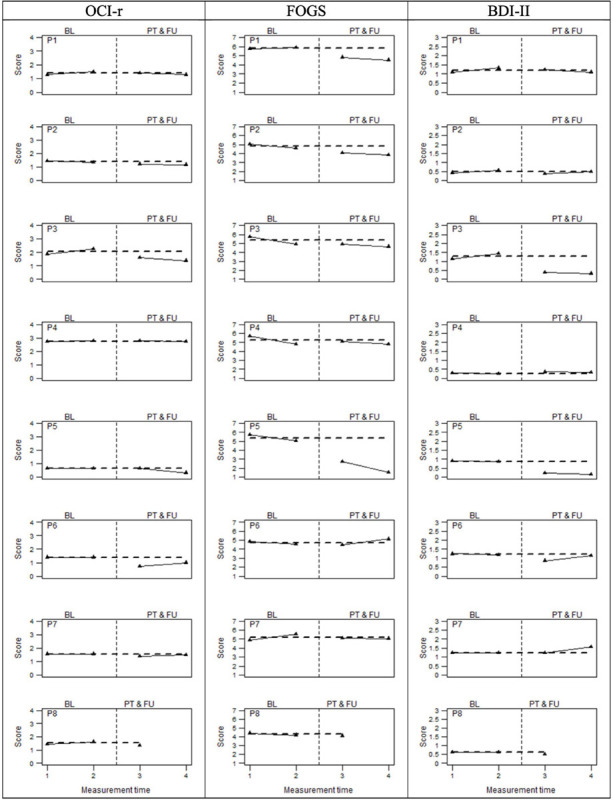
OCD related measures at baseline (BL), Post-treatment (PT), and follow-up (FU). The perpendicular dotted line represents the intervention.

As regards the secondary outcomes, Figure 3 graphically displays the OCD-related symptoms’ severity assessed by means of the OCI-r and two other measures linked to OCD, namely, the FOGS and the BDI-II. In this case, visual inspection suggested not entirely consistent improvement for all participants on the measures of interest. Although a score reduction could be observed for most participants on the OCI-r, FOGS, and BDI-II, these reductions did not seem to be as large as the Y-BOCS improvements. Non-overlapping data analyses (Table 4) confirmed these visual observations, indicating overall medium beneficial intervention effects (i.e., around 85%) on OCD symptoms severity (OCI-r) and fear of guilt (FOGS), while indicating a small effect (i.e., around 68%) on depression (BDI-II). Also, the analyses pertaining to individual change score highlighted a similar pattern of results (see Supplementary Table 1). With regard to the OCI-r, we detected a reliable improvement only for participants 3, 5, and 6 between pre-treatment measurement time, post-treatment, and follow-up phases. Moreover, participant 3 also showed a reliable worsening within the baseline phases which, if associated with the improvement of the scores in the post-treatment phases, indicates a real turnaround. The other participants demonstrated a reduction of symptom severity even if no reliable improvements were observed for them when comparing these phases. Similar results emerged on the measure of fear of guilt (i.e., FOGS). We observed a reliable improvement for participants 1, 2, 5, and 7. The remaining four participants reported improvements, but they were not reliable, while participant 6 showed a reliable deterioration in the follow-up. For five participants, baseline scores were not stable and they had already begun to decrease pre-treatment. This highlighted a trend that was partially independent of the treatment. However, considering the percentage of data points exceeding the median (PEM), which takes into account the autocorrelation of the scores in the different phases, a large and beneficial effect (i.e., 93%) of the intervention on fear of guilt clearly emerged. On the BDI-II, all participants demonstrated stable or worsening depression during the baseline measurement interval. After the intervention, six participants reported pre- to post-treatment decreases in depression that did not overlap with baseline scores. However, only participants 3 and 5 showed an improvement that could be considered reliable in both the post-treatment and follow-up compared to the baseline. Participants 2 and 6 appeared to be reliably improved immediately post-intervention, whereas they did not report either improvement or worsening at the follow-up. Participant 1 instead showed a reliable decrease in depression at both post-treatment and follow-up, but only the latter could be considered reliable. Participant 5 was the one who benefitted the most from treatment, showing stable scores at the baselines and a reliable improvement after the intervention. Participants 4 and 7 displayed a non-reliable worsening in the post-treatment phase, with P7 showing a reliable deterioration at the follow-up. Participant 8 showed improvements, but not reliably across phases.

**FIGURE 4:**
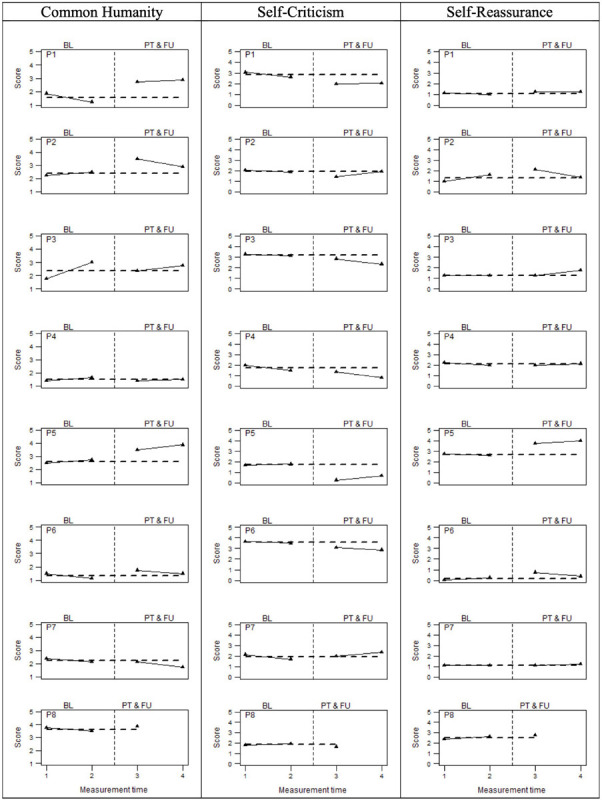
Compassion related measures at baseline (BL), Post-treatment (PT), and follow-up (FU). The perpendicular dotted line represents the intervention.

Finally, Figure 4 graphically displays individual compassion-related scores assessed by means of the common humanity subscale (CHS) and self-criticism and self-reassurance (FSCRS subscales). For this latter scale, the sub-dimensions of inadequate and hated self were investigated as a unitary dimension (i.e., self-criticism), while the self-reassurance dimension was examined separately. Visual inspection of the data suggested that five participants showed a visible score increase in common humanity, whereas four participants did on the self-reassurance dimension. Six participants displayed a decrease in self-criticism. As shown in Table 4, the three percentage indicators of data non-overlapping (i.e., PAND, NAP, and PEM) were around 74% for common humanity, 80% for the self-reassurance, and about 87% for self-criticism, suggesting medium/large beneficial effects of the compassion-focused intervention.

Five participants (i.e., P1, P2, P5, P6, and P8) displayed a reliable improvement in common humanity, both comparing pre- to post-treatment and pre- to follow-up measurements. In turn, three of these participants evidenced stability (i.e., P8) or reliable worsening (i.e., P1 and P6) at the baselines, which substantiated their improvement. Contrastingly, participants 2 and 5 demonstrated a reliable improvement already within the prescreening observations, which indicated a trend that was partially independent of the treatment. Participants 3 and 7 did not reveal any benefits due to the intervention, showing a reliable worsening in the post-treatment phase and in the follow-up, respectively. As regards self-criticism, all participants reported a reliable improvement, except for P7 who showed a reliable worsening both at post-treatment and follow-up. Four of them (i.e., P2, P5, P6, and P8) also reported stable scores within the baseline, whereas the other three (i.e., P1, P3, and P4) already demonstrated a reliable improvement. Finally, we observed an improvement on the self-reassurance dimension for all the eight participants involved in the study. For three of them (i.e., P2, P5, and P6), these improvements could be considered reliable comparing the pre- to post-treatment, whereas for participants 3 and 5, they could be considered reliable between the pre-treatment and the follow-up. Moreover, none reported a worsening on self-reassurance after the compassion-focused intervention. Therefore, self-criticism and self-reassurance represent the compassion-related measures where we observed the greatest effect of the compassion-focused intervention.

### Wilcoxon Signed Ranks Test

Given the small number of participants, we tried to provide further support and robustness to our results through another non-parametric test. Thus, we implemented a Wilcoxon Signed Ranks Test for the non-parametric comparison of the investigated constructs between the pre-compassion-focused intervention baseline, the post-intervention, and follow-up. Results were consistent with those of the non-overlapping data analyses (see Table 5). As regards the primary outcome, the Wilcoxon tests showed a significant decrease of the scores on the Y-BOCS and the related subdimensions both at the post-treatment and at the follow-up. Moreover, all test statistics were associated with a high effect size (*r*). As had emerged in the previous analyses, a unique exception was represented by a score on the compulsive dimension at the follow-up period, which was ultimately only marginally significant. Indeed, one participant (i.e., P7) reported an increase of the score at that measurement time. As regards the secondary outcomes, the Wilcoxon tests also showed a significant decrease of the scores on the OCD symptoms’ severity assessed by means of the OCI-r. Thus, six participants’ scores reported decreases at the post-intervention and seven at the follow-up, indicating a significant treatment effect. Also, in this case, test statistics were accompanied by medium high effects size. With regard to FOGS and BDI-II, we found only a marginally significant treatment effect (*p* = 0.06) for the comparison between the pre- and post-intervention scores. For both measures, six participants showed an improvement at this interval. No significant effect emerged at the follow-up measurement time, though five participants reported an improvement. This non-significant effect was due to the fact that on the FOGS and BDI-II, respectively, one and two participants demonstrated a deterioration in the follow-up.

**TABLE 5:** Wilcoxon Signed Ranks Test for study variables.

	**Comparisons**	**Negative ranks**	**Positive ranks**	**Ties**	**Total**	***Z***	***p***	**Observations**	***r***
Y-BOCS	Pre vs. Post	8	0	0	8	–2.52	0.01	16	–0.63
	Pre vs. F-up	7	0	0	7	–2.37	0.02	14	–0.63
Obses.	Pre vs. Post	8	0	0	8	–2.53	0.01	16	–0.63
	Pre vs. F-up	7	0	0	7	–2.39	0.02	14	–0.64
Comp.	Pre vs. Post	8	0	0	8	–2.53	0.01	16	–0.63
	Pre vs. F-up	6	1	0	7	–1.78	0.07	14	–0.48
OCI-r	Pre vs. Post	6	1	1	8	–2.21	0.03	16	–0.55
	Pre vs. F-up	7	0	0	7	–2.37	0.02	14	–0.63
FOGS	Pre vs. Post	6	1	1	8	–1.86	0.06	16	–0.46
	Pre vs. F-up	5	1	1	7	–1.57	0.11	14	–0.42
BDI-II	Pre vs. Post	6	1	1	8	–1.86	0.06	16	–0.46
	Pre vs. F-up	5	2	0	7	–1.83	0.24	14	–0.48
CHS	Pre vs. Post	2	5	1	8	1.61	0.11	16	0.40
	Pre vs. F-up	3	4	0	7	1.19	0.23	14	0.32
S-C	Pre vs. Post	7	1	0	8	–2.11	0.03	16	–0.53
	Pre vs. F-up	5	2	0	7	–1.61	0.11	14	–0.43
S-R	Pre vs. Post	0	5	3	8	2.03	0.04	16	0.51
	Pre vs. F-up	1	6	0	7	1.62	0.10	14	0.43

*YBOCS, Yale–Brown Obsessive–Compulsive Scale, and the obsessive (Obses.) and compulsive (Comp.) sub-dimensions, OCI-r, Obsessive–Compulsive Inventory Revised; FOGS, Fear of Guilt Scale; BDI-II, Beck Depression Inventory II; CHS, Common Humanity Subscale; S-C, Self-Criticism; S-R, Self-Reassurance. Table reports comparisons between the baseline (BL) and pre-treatment (Pre), pre-treatment and post-treatment (Post), pre-treatment (Pre), and follow-up (F-up).*

Consistently with overlap data analyses, we found a non-significant effect on common humanity. Although five and four participants showed improvements in the post-treatment and follow-up phases, respectively, we also witnessed two and three participants who reported negative score at such phases. This limited the possibility of achieving statistical significance in this test. Finally, we found significant treatment effects for both self-criticism and self-reassurance regarding the comparison between the pre-test and the post-test. Here, seven out of eight participants showed improvement in self-criticism, and five in self-reassurance. Furthermore, both of these results were associated with a medium-high effect size. However, these effects were not consistent in the comparison between pre-treatment and follow-up.

### Hedges’ *G* Effect Size for Single-Case Designs

Standardized mean differences (*G* with 95% CIs) were calculated to estimate the overall magnitude of the intervention effect across participants. Table 6 presents the *G*-statistic comparing post-treatment vs. baseline and follow-up vs. baseline. As regards the primary outcome, these effect sizes suggest that across participants, the compassion-focused intervention was associated with large decreases in OCD-related symptom severity assessed with the Y-BOCS. As regards the secondary outcomes, medium effect size decreases at the OCI-r were observed. Moreover, a large effect size was observed also for the fear guilt (i.e., FOGS), whereas medium effect sizes were noted for the BDI-II and compassion-related constructs. As mentioned in the “Data Analyses” Section, for each effect size, a *Z* test statistic and related 95% CIs were computed. Thus, all these effects turned out to be significant following the two tailed *Z* distribution, except for that related to the Common Humanity measure at the follow-up.

**TABLE 6:** Shadish et al.’s (2014) *G* effect size with 95% CIs.

					**95% CI**	
**Measure**	**Comparisons**	***G***	**SE**	***Z***	**Lower**	**Upper**
Y-BOCS	BL vs. Post	–1.10	0.36	–3.07	–1.77	–0.39
	BL vs. F-up	–1.07	0.39	–2.73	–1.85	–0.31
Obses.	BL vs. Post	–1.29	0.41	–3.12	–2.10	–0.48
	BL vs. F-up	–1.28	0.45	–2.75	–2.18	–0.38
Comp.	BL vs. Post	–0.87	0.29	–3.03	–1.43	–0.31
	BL vs. F-up	–0.78	0.29	–2.71	–1.35	–0.22
OCI-r	BL vs. Post	–0.29	0.13	–2.28	–0.55	–0.04
	BL vs. F-up	–0.34	0.15	–2.26	–0.63	–0.05
FOGS	BL vs. Post	–1.00	0.36	–2.78	–1.71	–0.29
	BL vs. F-up	–1.12	0.40	–2.79	–1.90	–0.33
BDI-II	BL vs. Post	–0.56	0.21	–2.72	–0.97	–0.16
	BL vs. F-up	–0.41	0.18	–2.24	–0.77	–0.05
CHS	BL vs. Post	0.49	0.25	2.02	0.01	0.97
	BL vs. F-up	0.57	0.31	1.83	–0.40	1.87
S-C	BL vs. Post	–0.29	0.12	–2.39	–0.52	–0.05
	BL vs. F-up	–0.63	0.25	–2.79	–1.13	–0.13
S-R	BL vs. Post	0.35	0.14	2.38	0.06	0.63
	BL vs. F-up	0.32	0.15	2.12	0.02	0.62

*Table reports standardized mean differences between the average scores at the baseline vs. post-treatment and vs. follow-up scores. Test statistics and confidence intervals follow the two-tailed *Z* distribution.*

### Acceptability

Quantitative feedback on the intervention revealed a high level of satisfaction with CFT-OCD. On a seven-point Likert scale (from 1 = not at all useful to 7 = extremely useful), mean scores of usefulness for the different aspects of the intervention for each item were all above 5.5, with practices in pairs, visualization techniques, and psychoeducation scoring the highest among the other elements of the intervention. On a five-point Likert scale (from 1 = not at all to 5 = extremely), mean scores of the acceptability of and satisfaction with the intervention were *M* = 4.25 (*SD* = 0.7) and *M* = 4.1 (*SD* = 0.7), respectively. It was also extremely likely that they would recommend this treatment to friends or family suffering from OCD, *M* = 4.5 (*SD* = 0.5), and they felt that the treatment had improved their quality of life, *M* = 4.4 (*SD* = 0.4). Informal feedback collected during the post-intervention assessment revealed that a number of clients reported developing a new relationship to their OCD during the treatment, seeing it as just a “scared and suffering” part of them that they could learn to treat with validation and compassion to maximize their resilience. They reported that they could use compassion as a “new language” to talk to their fears, but that it needed to be practiced (exactly like a language). The establishment of a positive, caring therapeutic relationship with the facilitators and with the other members of the group was also one of the sources of their change. The handouts and exercises were viewed as constructive, although home-practice felt at times distressing to some clients. Areas to improve in the protocol identified by clients included reshaping some handouts to be more structured, and further highlighting strategies to facilitate social reconnection and to deal with intrusive thoughts. One client (P4), whose depression slightly worsened during the treatment, still gave encouraging feedback about the treatment, identifying clear gains he/she had made, especially linked to the group climate.

## Discussion

This study is the first known study to evaluate the feasibility, acceptability, and potential clinical effectiveness of an 8-week compassion-focused intervention designed to reduce OCD symptoms and OCD-related constructs (fear of guilt, depression, and self-criticism), and increase compassionate self-reassurance and common humanity in a group of treatment-resistant OCD patients, using a multiple baseline design. Results showed preliminary but nevertheless promising evidence of clinical effectiveness on the primary outcome measure, substantiated by triangulation in findings across analytic methods.

In line with previous research suggesting the potential benefits of compassion-focused interventions for OCD (Barcaccia et al., 2015; Key et al., 2017; Chase et al., 2019; Didonna et al., 2019; Leeuwerik et al., 2020), this study found that all participants experienced a significant decrease in OCD symptoms as measured by the clinician-administered Y-BOCS, with large effect sizes. More specifically, during the two baseline assessments (T1 and T2), OCD symptoms remained stable or increased for 7/8 participants, irrespective of baseline duration. The only exception was participant P6, who showed a slight but reliable improvement during the two pre-treatment assessments (T1 and T2), but was still retained in the analysis given that the scores at the OCI-r did not corroborate such improvement. At post-intervention assessment (T3), 100% of participants demonstrated marked, reliable reductions in OCD symptoms, relative to their scores at baseline. Gains were mostly preserved at follow-up (T4), where 6/8 participants showed reliable reductions at Y-BOCS, relative to T2. One participant (P7) did not show improvements at follow-up. A more fine-grained analysis of the subdimensions of the Y-BOCS (obsessions and compulsions) confirmed the results: all participants reported a reliable improvement on the compulsions dimension of the Y-BOCS after the treatment. Only P7 showed a reliable worsening in compulsions (though also a reliable improvement on the obsessions) at the follow-up. Regarding the obsessions dimension, P4 was the only participant who did not highlight any improvement across phases (though there was a reliable improvement on the compulsion dimension).

The beneficial effects of the treatment on the severity of OCD symptoms were confirmed by changes on the self-administered OCI-r, which was improved in 85% of the participants. The stable or worsening baselines, the decrease in OCD symptoms only after the intervention was introduced, and the magnitude of the changes indicate that the intervention effect is not likely due to repeated assessments, self-monitoring, the passage of time, chance fluctuations, regression to the mean, or spontaneous recovery.

In trying to explain the worsening in compulsions at follow-up of P7, it is important to note that the T3 and T4 data collection were carried out, respectively, right after the announcement of the Italian lockdown due to the novel coronavirus pandemic (March 9, 2020) and after 1 month (i.e., right in the middle of the lockdown). As follows, this may have exacerbated the symptoms related to contamination, illness, and concern about accidental harm (Conrad et al., 2020), undermining the effectiveness of the intervention especially for P7, who nonetheless showed improvements in obsessions. Thus, the CFT intervention might have provided long-term resilience against mental intrusions, but not against the automaticity of compulsory behaviors (checking, washing, etc.), which, at the time of follow up, were indeed encouraged and made mandatory by the Italian government.

Interestingly, improvements at post-test were shown by all patients irrespective of the type of OCD they presented. This suggests that the CFT-OCD intervention, in line with its evolutionary transdiagnostic nature (Gilbert, 2014) has hit a core element that is shared by different types of OCD presentations. Such a significant improvement in OCD symptoms shown by all participants is a notable finding, given that none of the sessions of the intervention entailed ERP procedures, which are considered to be the first-line treatment for OCD symptoms (Wheaton et al., 2015). It is generally thought that ERP is necessary for practicing a new, more adaptive response to anxiety-provoking stimuli and for substantially improving the prognosis of OCD (Öst et al., 2015). However, although several studies have found large improvements in OCD symptoms after ERP, the outcomes are sub-optimal for the majority of patients (60% of treatment completers achieve recovery, and approximately 25% of patients are asymptomatic following treatment; Fisher and Wells, 2005). Furthermore, 30% of patients with OCD refuse ERP or drop out from treatment prematurely, suggesting that ERP might be difficult to tolerate (Whittal et al., 2005). The results of the present pilot study suggest that a relatively brief group compassion-focused intervention may be effective for reducing OCD symptoms, even without deliberately implementing ERP procedures. Future studies will have to evaluate the differential efficacy and retention rates of introducing compassion-focused intervention before ERP, and explicitly applying compassion-focused skills to ERP.

The results of the other secondary outcomes in our study might shed some light on the mechanisms involved in the improvement of OCD symptoms reported by almost all participants. In line with our expectation, the majority of participants experienced pre- to post-treatment decreases in self-reported symptoms of depression, but overall findings suggest small-to-medium beneficial effects of the compassion-focused intervention. This might be due to the worsening in depression at the post-treatment phase reported by one participant (P7). Given that the same participant also showed a reliable worsening in compulsions (but a reliable improvement in obsessions) at the follow-up, it is plausible to assume that this deterioration in depressive symptoms might not be linked to the intervention, but to external precipitating circumstances (the pandemic lockdown in Italy). These findings are consistent with previous studies investigating the benefits of CFT for depressive symptoms (i.e., Frostadottir and Dorjee, 2019) and suggest that the adapted CFT for OCD intervention may target both OCD symptoms and symptoms of depression. At the same time, it seems that an improvement in depression was not the driving force behind OCD symptoms’ reduction in the majority of participants.

In line with our expectations, a large and beneficial effect of the intervention emerged on the measure of fear of guilt. There was a reduction in symptom severity in 81% of the participants, indicating a significant treatment effect. Moreover, even though some of the participants’ baseline scores already started to decrease before the intervention, the percentage of data points exceeding the median (PEM) was 93%, showing a large effect. This finding is encouraging, given the increasingly recognized centrality of fear of guilt in the etiology and maintenance of OCD (Shapiro and Stewart, 2011; Mancini, 2019). However, only few studies have tested the effectiveness of psychological interventions intended to directly impact fear of guilt, and most have used cognitive procedures like socratic dialogue, cognitive restructuring, and double standard (Cosentino et al., 2012; Perdighe and Mancini, 2012). This study provides the first empirical evidence that fear of guilt can be reduced not only by targeting it directly (asking patients not to prevent the guilt, but to expose themselves to it), but also by increasing patients’ capacity to develop a compassionate attitude toward themselves (and parts of themselves, including the fear of guilt) and others.

In line with our predictions, all participants except one (P7) showed a reduction in self-criticism, and we observed an improvement in compassionate self-reassuring for all eight participants involved in the study. These findings were consistent with previous investigations of compassion-focused interventions that have been found effective in improving the self-to-self relationship (increased self-compassion and reduced self-criticism) in non-clinical samples (i.e., Matos et al., 2017; Sommers-Spijkerman et al., 2018) and in different clinical populations (Au et al., 2017; Heriot-Maitland et al., 2019; Fox et al., 2020). Findings of the present pilot study suggest that this type of intervention is effective also in improving compassionate self-reassurance and self-criticism in treatment-resistant OCD patients. They also seem to suggest potential mechanisms driving the positive change reported in OCD symptoms.

It is possible that compassion-focused practices have helped participants strengthen their capacity to build and access compassionate self-reassuring skills, dampening their chronically over-stimulated threat system and facilitating an improved physiological regulation of their overall arousal. As we know, chronically increased negative arousal modulates information processing, prompting a switch from a context-based and flexible cognitive system to a more rigid safety-focused cognitive processing, characteristic of OCD patients (Maran et al., 2018; Mancini, 2019). Instead, the switch from an avoidance/safety-focused motivation to an approach/care-focused motivation (i.e., compassion both for ourselves and others), cultivated in CFT treatments, might have promoted a felt sense of *safeness*, with resulting improved cognitive flexibility and ability to tolerate emotional disturbance (Petrocchi and Cheli, 2019).

Another possible mechanism of change is linked to the creation of a compassionate “*inner secure base and safe haven”* (Gilbert, 2017) that might have helped participants explore, face, and accept the “humanness” of making mistakes and, at times, experiencing guilt. As one participant reported: “when my doubts arrive, and I start sinking into my spirals of fear and I’m tempted to go and do my stuff (i.e., *washing*), now I know that I can ask for the help of my compassionate image…visualize it close to me. I see her smile, I imagine her scent of coconut, and her voice whispering… *I know what you are going through*…*but it’s not your fault. I know it’s hard, but you’re not alone, I’ve been there before you and now I’m here with you*…*we can stay, you’re not alone.* In her presence I see the comforting message that I, too, like everyone can afford my doubts…I’m big enough, I’m spacious enough to embrace them… I don’t have to escape from that ocean because now I have my compassionate life-jacket that can help me navigate those dark waters….” As anecdotally reported in this comment, and suggested by the reduced fear of guilt participants showed, CFT practices seemed to promote an improved acceptance (not an avoidance) of threat (both internal, such as feelings of guilt, and external, such as people scolding or potentially rejecting). CFT practices also seemed to reduce (instead of increase) the typical excessive reassurance seeking that is unequivocally counter-productive in people suffering from anxiety disorders (OCD in particular), yielding short-term relief but a longer-term worsening of the original anxiety (Salkovskis and Kobori, 2015). However, further studies will have to confirm this observation.

Surprisingly, the intervention indicated non-completely coherent improvements on the dimension of common humanity, where 74% of participants improved while three participants worsened at post-intervention and follow-up (P3, P4, and P7). It is possible that the group intervention was too short (8 weeks) to generate a deep sense of inter-connection in all participants. In particular, it is possible that P4 and P7, who did not improve in both dimensions of Y-BOCS like other participants, might have perceived the improvement of others as even more isolating (“I’m the only one who does not improve”). Research is needed regarding moderators (both personality traits and amount of change during the treatment itself) of successful CFT interventions with OCD patients. Nonetheless, this result seems to indicate that improvements in OCD symptoms seem not to be overly connected to a general and non-specific sense of common humanity that resulted from the participation in a group intervention.

The increased ability to dampen self-criticism and activate a compassionately reassuring inner self that accepts, validates, and is willing to soothe painful emotions (such as guilt) seemed to be the active ingredient of the intervention. Indeed, even if the limited sample in this study prevented the use of mediational analysis, a purely explorative correlation analysis at group level among change scores (T3 *minus* T2) showed a marginally significant correlation between improvements in self-reassurance and Y-BOCS (*r* = −0.68; *p* = 0.06), and significant correlation between improvements in fears of guilt and self-reassurance (*r* = −0.83; *p* = 0.01), and between improvements in fears of guilt and self-criticism (*r* = 0.84; *p* = 0.01). However, caution should be used in interpreting these exploratory findings, since the study design did not permit direct comparison of different treatment components. Further investigation is needed to explore the theorized potential mechanisms for change in CFT-OCD, using a larger sample and mediational analyses.

The secondary aim of the study was to assess the feasibility and acceptability of CFT-OCD and feedbacks from participants revealed a high level of satisfaction with it. Core elements of the interventions were all rated as very useful (mean scores of 5.5 out of 7), with practices in pairs, visualization techniques, and psychoeducation scoring the highest. Participants reported that CFT-OCD was acceptable, that they were satisfied with it, and that they would recommend it to friends and family. Given the high dropout rates in individual CBT for OCD (estimated to be greater than 20%; Ong et al., 2016), the retention rates in the case series are encouraging. In fact, all participants completed the intervention. This strong retention rate provides further support that participants found the intervention acceptable and feasible.

There are a number of limitations that need to be held in mind regarding the present findings.

First, the absence of an active control condition makes it impossible to discriminate between the specific CFT-DOC effect and any non-specific effects of therapy. In addition, the lead author was the treatment developer and one of the facilitators: thus, it is not possible to rule out therapist-specific effects and demand characteristics. The absence of assessments during the active phase of the intervention did not allow us to assess changes at each session and makes it impossible to evaluate the efficacity of specific elements of the intervention. Additionally, we did not collect home-practice logs, and it was not possible to estimate the impact of home practice on the reported improvements. Though we did not rely solely on self-report measures, we used a limited number of compassion-related measures as we did not want to overburden our participants. Future studies should evaluate the intervention against an active control condition, include independent therapists and ratings of therapist rapport and competency, and use additional compassion-related measures to explore potential mechanisms of change more in depth. The study is also limited in its ability to generalize the findings from a small sample to diverse populations; all participants were well educated, relatively young, and high-functioning. A critical future direction would be to test the intervention for more diverse samples and OCD types. In particular, it will be important to examine if treatment response varies as a function of intake OCD severity.

Finding from this pilot study provide preliminary evidence that CFT is associated with reductions in OCD symptoms in treatment-resistant patients. The brief nature of this intervention and the improvements observed indicate that it may be promising as either a stand-alone treatment or as an adjunct to other treatments. Even though results in some variables were not always reliable for all variables (for example, depression and common humanity) and across the different phases, we believe that they are noteworthy. Additionally, it should be taken into account that the last two data collections (i.e., the post-treatment and the follow-up) were carried out right at the beginning and in the middle of the Italian lockdown. This may have somehow altered the mental stability of participants, undermining the effectiveness of compassion-focused intervention, which nevertheless withstood the hit.

## Data Availability Statement

The raw data supporting the conclusions of this article will be made available by the authors, without undue reservation.

## Ethics Statement

The studies involving human participants were reviewed and approved by Università Guglielmo Marconi – Roma. The patients/participants provided their written informed consent to participate in this study.

## Author Contributions

NP, TC, and FM contributed to conception and design of the study. NP and AD’I conducted the intervention. GF conducted all the assessments. GF and AD’I organized the database. VP performed the statistical analysis. NP and VP wrote the first draft of the manuscript. All authors contributed to manuscript revision, and read and approved the submitted version.

## Conflict of Interest

TC, GF, AD’I, and FM were employed by company Scuola di Psicoterapia Cognitiva S.r.l., Rome, Italy. The remaining authors declare that the research was conducted in the absence of any commercial or financial relationships that could be construed as a potential conflict of interest.
