# Supplementary material for: Compassion-Focused Group Therapy for Treatment-Resistant OCD: Initial Evaluation Using a Multiple Baseline Design
Source: Front Psychol. 2021 Jan 12;11:594277. doi: 10.3389/fpsyg.2020.594277 (PMC7835278; doi:10.3389/fpsyg.2020.594277)
Supplement: Supplementary file 1 [file Table_1.DOCX]

Supplementary Material

# Supplementary Table 1. Change score and 95% CIs of the measures in the study

|  | **Y-BOCS** | **Obses.** | **Comp.** | **OCI-r** | **FOGS** | **BDI-II** | **CHS** | **FSCRS** | |
| --- | --- | --- | --- | --- | --- | --- | --- | --- | --- |
|  |  |  |  |  |  |  |  | **S-C** | **S-R** |
| **P1** |  |  |  |  |  |  |  |  |  |
| BL-Pre | 0 | 0 | 0 | 0.54 | 0.29 | 0.96^ | -1.85^ | -1.28* | -0.34 |
|  | (-0.45, 0.45) | (-0.66, 0.66) | (-0.42, 0.42) | (-0.27, 1.34) | (-0.53, 1.11) | (0.49, 1.43) | (-2.52, -1.19) | (-1.94, -0.62) | (-1.09, 0.41) |
| Pre-Post | -2.63* | -1.19* | -3.67* | -0.27 | -2.54* | -0.38 | 4.41* | -1.91* | 0.65 |
|  | (-3.08, -2.18) | (-1.85, -0.53) | (-4.10, -3.25) | (-1.07, 0.53) | (-3.36, -1.72) | (-0.85, 0.09) | (3.75, 5.08) | (-2.57, -1.25) | (-0.09, 1.40) |
| Pre-FU | -2.19* | -0.59 | -3.67* | -0.54 | -3.23* | -0.96* | 4.80* | -1.70* | 0.65 |
|  | (-2.64, -1.74) | (-1.25, 0.07) | (-4.10, -3.25) | (-1.34, 0.27) | (-4.05, -2.42) | (-1.43, -0.49) | (4.13, 5.46) | (-2.36, -1.04) | (-0.09, 1.40) |
| **P2** |  |  |  |  |  |  |  |  |  |
| BL-Pre | 0 | 0 | 0 | -0.27 | -0.13* | 0.58^ | 0.74* | -0.63 | 1.65* |
|  | (-0.45, 0.45) | (-0.66, 0.66) | (-0.42, 0.42) | (-1.07, 0.54) | (-1.94, -0.31) | (0.11, 1.05) | (0.07, 1.40) | (-1.28, 0.03) | (0.90, 2.40) |
| Pre-Post | -5.26* | -2.37* | -6.43* | -0.27 | -1.27* | -0.79* | 2.94* | -1.28* | 1.31* |
|  | (-5.70, -4.81) | (-3.03, -1.71) | (-6.86, -6.00) | (-1.07, 0.54) | (-2.09, -0.45) | (-1.26, -0.32) | (2.28, 3.61) | (-1.94, -0.62) | (0.56, 2.06) |
| Pre-FU | -6.57* | -2.97* | -8.26* | -0.39 | -1.85* | -0.38 | 1.12* | 0.21 | -0.65 |
|  | (-7.02, -6.12) | (-3.63, -2.31) | (-8.69, -7.84) | (-1.19, 0.41) | (-2.66, -1.03) | (-0.85, 0.89) | (0.45, 1.78) | (-0.45, 0.87) | (-1.40, 0.09) |
| **P3** |  |  |  |  |  |  |  |  |  |
| BL-Pre | -0.44 | 0.59 | -1.84* | 0.95^ | -1.96* | 1.21^ | 3.68* | -0.45* | 0 |
|  | (-0.89, 0.01) | (-0.07, 1.25) | (-2.26, -1.41) | (0.15, 1.75) | (-2.78, -1.15) | (-0.74, 1.68) | (3.01, 4.34) | (-1.10, -0.21) | (-0.75, 0.75) |
| Pre-Post | -3.07* | -2.97* | -1.84* | -1.63* | 0 | -4.38* | -1.82^ | -0.83* | 0 |
|  | (-3.51, -2.62) | (-3.63, -2.31) | (-2.26, -1.41) | (-2.44, -0.83) | (-0.82, 0.82) | (-4.85, -3.91) | (-2.49, -1.16) | -1.49, -0.18 | (-0.75, 0.75) |
| Pre-FU | -3.07* | -2.97* | -1.84* | -2.17* | -0.69 | -4.59* | -0.74^ | -2.33* | 1.31* |
|  | (-3.51, -2.62) | (-3.63, -2.31) | (-2.26, -1.41) | (-2.97, -1.37) | (-1.51, 0.12) | (-5.06, -4.12) | (-1.40, -0.07) | (-2.98, -1.67) | (0.56, 2.06) |
| **P4** |  |  |  |  |  |  |  |  |  |
| BL-Pre | 0 | 0 | 0 | 0.15 | -2.13* | -0.17 | 0.73* | -1.49* | -0.65 |
|  | (-0.45, 0.45) | (-0.66, 0.66) | (-0.42, 0.42) | (-0.66, 0.95) | (-2.95, -1.31) | (-0.64, 0.30) | (0.07, 1.41) | (-2.15, -0.08) | (-1.40, 0.09) |
| Pre-Post | -0.88* | -0.59 | -0.92* | 0 | 0.71 | 0.42 | -0.73^ | -0.42 | 0 |
|  | (-1.32, -0.43) | (-1.25, 0.07) | (-1.35, -0.49) | (-0.80, 0.80) | (-0.09, 1.54) | (-0.05, 0.89) | (-1.41, -.07) | (-1.07, 0.24) | (-0.75, 0.75) |
| Pre-FU | -0.88* | -0.59 | -0.92* | -0.15 | 0 | 0.33 | -0.38 | -2.11* | 0.34 |
|  | (-1.32, -0.43) | (-1.25, 0.07) | (-1.35, -0.49) | (-0.95, 0.66) | (-0.82, 0.82) | (-0.14, 0.80) | (-1.05, 0.28) | (-2.77, -1.46) | (-0.41, 1.09) |
| **P5** |  |  |  |  |  |  |  |  |  |
| BL-Pre | 0 | 0 | 0 | 0 | -1.56* | -0.17 | 0.73* | 0.30 | -0.31 |
|  | (-0.45, 0.45) | (-0.66, 0.66) | (-0.42, 0.42) | (-0.80, 0.80) | (-2.37, -0.74) | (-0.64, 0.30) | (0.07, 1.40) | (-0.36, 0.96) | (-1.06, 0.43) |
| Pre-Post | -3.94* | -4.75* | -2.75* | 0 | -5.63* | -2.59* | 2.21* | -4.47* | 2.93* |
|  | (-4.39, -3.50) | (-5.41, -4.09) | (-3.18, -2.33) | (-0.80, 0.80) | (-6.45, -4.81) | (-3.06, -2.12) | (1.54, 2.87) | (-5.12, -3.81) | (2.18, 3.68) |
| Pre-FU | -5.70* | -4.75* | -4.59* | -0.83* | -8.45* | -2.96* | 3.32* | -3.22* | 3.59* |
|  | (-6.14, -5.25) | (-5.41, -4.09) | (-5.02, -4.16) | (-1.63, -0.03) | (-9.27, -7.64) | (-3.43, -2.49) | (2.66, 3.99) | (-3.88, -2.56) | (2.84, 4.34) |
| **P6** |  |  |  |  |  |  |  |  |  |
| BL-Pre | -0.88* | -1.19* | 0 | 0 | -0.83* | -0.21 | -1.09^ | -0.42 | 0.64 |
|  | (-1.32, -0.43) | (-1.85, -0.53) | (-0.42, 0.42) | (-0.80, 0.80) | (-1.65, -0.21) | (-0.68, 0.26) | (-1.75, -0.42) | (-1.07, 0.24) | (-0.09, 1.40) |
| Pre-Post | -5.70* | -2.97* | -6.43* | -1.63* | -0.14 | -1.38* | 1.82* | -1.28* | 1.31* |
|  | (-6.14, -5.25) | (-3.63, -2.31) | (-6.86, -6.00) | (-2.44, -0.83) | (-0.96, 0.67) | (-1.85, -0.91) | (1.15, 2.49) | (-1.90, -0.62) | (0.56, 2.06) |
| Pre-FU | -6.57* | -3.56* | -8.26* | -0.95* | 1.41^ | -0.21 | 1.09* | -1.91* | 0.34 |
|  | (-7.02, -6.12) | (-4.22, -2.90) | (-8.69, -7.84) | (-1.75, -0.15) | (0.59, 2.23) | (-0.68, 0.26) | (0.42, 1.75) | (-2.56, -1.25) | (-0.41, 1.09) |
| **P7** |  |  |  |  |  |  |  |  |  |
| BL-Pre | -0.44 | -0.59 | 0 | 0 | 1.56^ | 0 | -0.74^ | -1.28* | 0 |
|  | (-0.89, 0.01) | (-1.25, 0.07) | (-0.42, 0.42) | (-0.80, 0.80) | (0.74, 2.37) | (-0.47, 0.47) | (-1.40, -0.07) | (-1.93, -0.62) | (-0.75, 0.75) |
| Pre-Post | -1.31* | -0.59 | -1.84* | -0.41 | -0.98* | 0 | 0 | 0.86^ | 0 |
|  | (-1.76, -0.87 | (-1.25, 0.07) | (-2.26, -1.41) | (-1.22, 0.39) | (-1.80, -0.16) | (-0.47, 0.47) | (-0.67, 0.67) | (0.21, 1.52) | (-0.75, 0.75) |
| Pre-FU | -0.44 | -2.97* | 3.67^ | -0.15 | -1.13* | 1.37^ | -1.12^ | 1.94^ | 0.31 |
|  | (-0.89, 0.01) | (-3.63, -2.31) | (3.25, 4.10) | (-0.95, 0.66) | (-1.94, -0.31) | (0.91, 1.85) | (-1.78, -0.45) | (1.28, 2.59) | (0.43, 1.06) |
| **P8** |  |  |  |  |  |  |  |  |  |
| BL-Pre | 0.88^ | 0 | 1.84^ | 0.41 | -0.55 | 0 | -0.73 | 0.42 | 0.65 |
|  | (0.43, 1.32) | (-0.66, 0.66) | (1.41, 2.26) | (-0.39, 1.22) | (-1.36, 0.27) | (-0.47, 0.47) | (-1.40, 0.07) | (-0.24, 1.07) | (-0.09, 1.40) |
| Pre-Post | -4.38* | -4.15* | -2.75* | -0.68 | -0.14 | -0.42 | 1.12* | -0.86* | 0.31 |
|  | (-4.83, -3.93) | (-4.81, -3.49) | (-3.18, -2.33) | (-1.49, 0.12) | (-0.96, 0.67) | (-0.89, 0.05) | (0.45, 1.78) | (-1.52, -0.21) | (-0.43, 1.06) |

Note. Yale-Brown Obsessive-Compulsive Scale (YBOCS) and the obsessive (Obses.) and Compulsive (Comp.) sub-dimensions, Obsessive-Compulsive Inventory Revised (OCI-r), Fear of Guilt Scale (FOGS), Beck Depression Inventory II (BDI-II), Common Humanity Subscale(CHS), Self-Criticism (S-CA) and Self-Reassurance (S-R). Table reports comparisons between the Baseline (BL) and Pre-Treatment (Pre), Pre-Treatment and Post-Treatment (Post), Pre-Treatment (Pre) and Follow-up (FU). Each cell displays a change score. Negative change scores indicate decreases on a given measure, positive change scores indicate increases. * indicates reliable improvement. ^ indicates reliable worsening. Values ​​reported in parentheses refer to 95% Confidence Intervals.
